# Phosphate starvation enhances phagocytosis of *Mycobacterium bovis*/BCG by macrophages

**DOI:** 10.1186/s12865-020-00364-x

**Published:** 2020-06-09

**Authors:** Patricia Espinosa-Cueto, Alejandro Magallanes-Puebla, Raul Mancilla

**Affiliations:** Departamento de Inmunología, Instituto de Investigaciones Biomédicas, Universidad Nacional Autónoma de México, Circuito Escolar S/N, Ciudad Universitaria, 04510 México City, Mexico

**Keywords:** Tuberculosis, *M.bovis/*BCG, Vaccine, Adhesins, Macrophages

## Abstract

**Background:**

Tuberculosis is an important health problem worldwide. The only available vaccine is *M. bovis/BCG*, an attenuated mycobacterium that activates the innate and the acquired immune system after being phagocytosed by macrophages and dendritic cells. Vaccination fails to prevent adult pulmonary tuberculosis although it may have a protective effect in childhood infection. Understanding how BCG interacts with macrophages and other immunocompetent cells is crucial to develop new vaccines.

**Results:**

In this study we showed that macrophages phagocytose *M. bovis*/BCG bacilli with higher efficiency when they are cultured without phosphate. We isolated mycobacterial membranes to search for mycobacterial molecules that could be involved in these processes; by immunoblot, it was found that the plasma membranes of phosphate-deprived bacilli express the adhesins PstS-1, LpqH, LprG, and the APA antigen. These proteins are not detected in membranes of bacilli grown with usual amounts of phosphate.

**Conclusions:**

The interest of our observations is to show that under the metabolic stress implied in phosphate deprivation, mycobacteria respond upregulating adhesins that could improve their capacity to infect macrophages. These observations are relevant to understand how *M. bovis/*BCG induces protective immunity.

## Background

Tuberculosis (TB) remains an important health problem worldwide. In 2017, there were 10.4 million new cases and 1.7 million deaths [1]. Pulmonary TB is initiated when inhaled bacilli reach the alveoli, the terminal part of the respiratory tract, where they encounter host cells, especially macrophages (MO), the best-equipped cell to contend with intracellular microbes [2, 3]. After phagocytosis, mycobacteria can be lysed within the phagolysosome, although virulent bacilli have developed the ability to avoid destruction impeding the fusion of the phagosome with the lysosome, thus transforming the MO in a friendly niche where they proliferate and persist [2, 4]. In an initial step, *Mycobacterum tuberculosis* (Mtb) binds alveolar MOs through mycobacterial adhesins or opsonins that interact with a variety of receptors, including CR1, CR3, and CR4, IgGFc, surfactant A protein and C-type lectins [3]. Information on the repertoire of Mtb adhesins is scanty; a few have been characterized including lipoarabinomannan, a cell wall glycolipid [4], LpqH, a 19-kDa surface located lipoprotein [5], the heparin-binding hemagglutinin [6], PE_PGRS33, a member of the PE-PGRS family [7], LprG a 25-kDa glycolipoprotein [8], APA a 45–47-kDa mannose-containing glycoprotein [9] and PstS-1, a phosphate transporter [10]. It is important to know better the repertoire of mycobacterial adhesins since it could be helpful to design novel strategies for TB prevention and treatment [11].

Recently, it has been reported that PstS-1, the 38-kDa glycoprotein, binds the MO mannose receptor triggering the phagocytosis of mycobacteria [10]. PstS-1 has focused much interest as a phosphate (Pi) transporter whose expression is upregulated under Pi starvation conditions [12–14]. Pi deprivation can activate the expression of a variety of genes not involved in phosphate metabolism that participate in the pathogenesis of TB [15]. Herein, we show that *M. bovis*/BCG bacilli grown without phosphate are engulfed with increased efficiency by MO, an event that was associated with decreased phagosome acidification. Considering that Pi starvation increases the surface expression of PstS-1, we searched its expression in isolated plasma membranes; we found that together with PstS-1, the membranes of Pi-deprived mycobacteria express the mycobacterial adhesins LpqH, LprG, and the APA antigen, glycoproteins that are not directly involved in phosphate regulation.

## Results

### Mycobacteria cultured under Pi deprivation conditions are phagocytosed with higher efficiency by macrophages

**Fig. 1:**
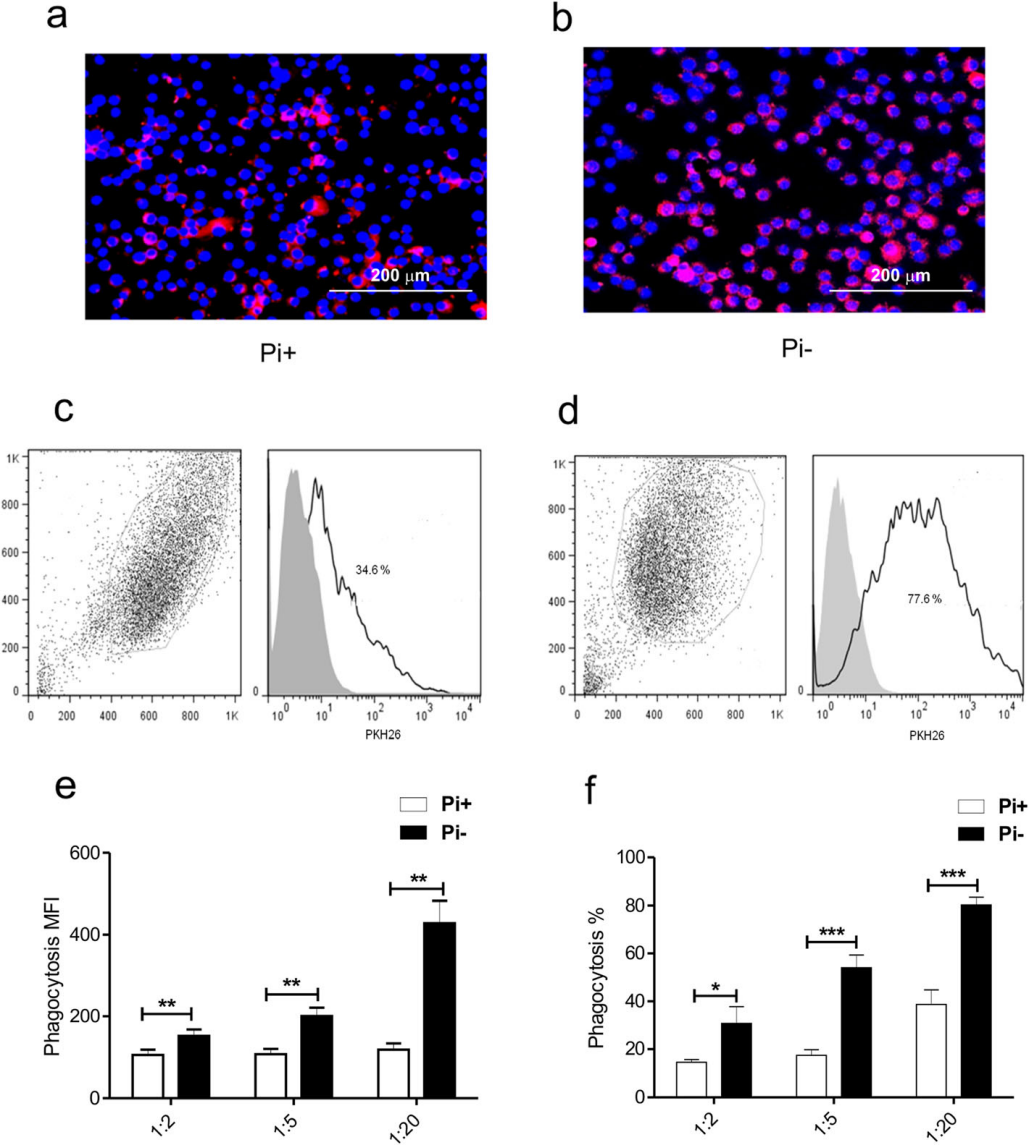
Phosphate deprivation increases *M. bovis*/BCG phagocytosis. J774A.1 MOs (5 × 10^5^) were incubated with *M. bovis*/BCG bacilli labeled red with the PKH-26; nuclei were stained blue with DAPI. Immunofluorescence microscopy shows a marked difference in the degree of phagocytosis between bacilli grown with and without Pi (**a**, **b**). For FACS analysis gates considering the percent and the mean fluorescence index (MFI) were carried out (**c**, **d**). Dose-dependent phagocytosis that was significantly higher with Pi-deprived mycobacteria was observed (**e**, **f**). At a 1:20 MOI, 80.2% of MOs phagocytosed bacilli cultured without Pi while the percent phagocytosis of bacilli cultured with Pi was 38.7; *p* < 0.05; unpaired t Student’s test. Results of 5 independent experiments are shown

The phagocytosis assays were carried out incubating J774A.1 macrophage-like cells with mycobacteria labeled with the lipophilic red fluorescent dye PKH-26 at 1:2, 1:5 and 1:20 MOI. Immunofluorescence microscopy revealed that the number of MOs with engulfed or bound bacilli was significantly higher with Pi-deprived mycobacteria (Fig. 1a, b). Phagocytosis was quantitated by fluorescence activated cell sorting (FACS) with PKH-26 stained bacilli (Fig. 1c, d). Phagocytosis expressed in mean fluorescence intensity (MFI) and percent was dose-dependent and considerably higher with Pi-deprived bacilli (Fig. 1e, f). At a 1:20 MOI, the percent of phagocytosis of bacilli grown without Pi was 80.2 and 38.7% of bacilli grown with usual amounts of Pi.

### Assays to verify the viability of phagocytosed mycobacteria grown with and without phosphate

**Fig. 2:**
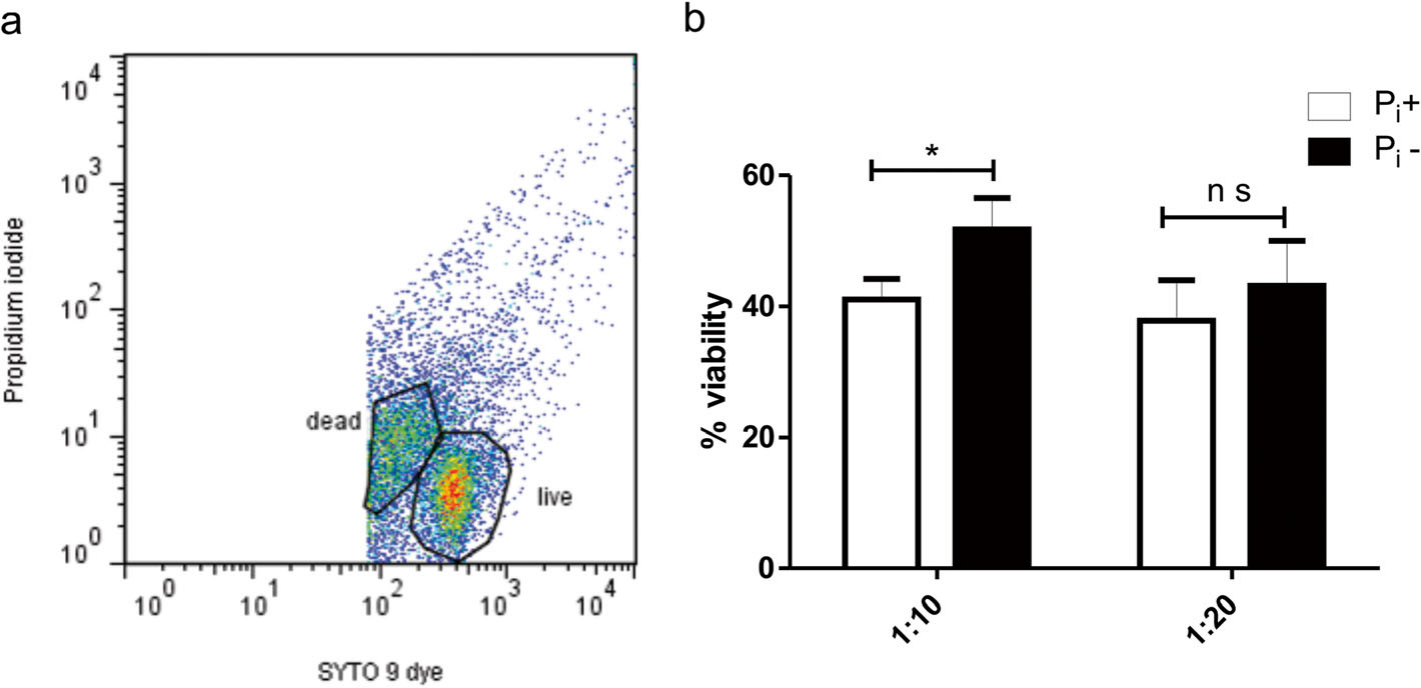
Phosphate deprivation increases the viability of mycobacteria within macrophages. After 4 h phagocytosis, mycobacteria viability was analyzed by FACS using the LIVE/DEAD viability assay. A dot plot was set to differentiate living from dead mycobacteria (**a**). The survival rate was calculated using the formula: % viability = # of bacteria in live region / # of bacteria in dead region x (100) (**b**). *p* < 0.05 Mann Whitney test. Results of 3 experiments are presented

After 4 h of MO phagocytosis at 1:10 and 1:20 MOI, the viability of ingested bacilli was assessed using the LIVE/DEAD Baclight bacterial viability Kit (Molecular Probes, Invitrogen, Carlsbad, CA EUA) following manufacturer’s instructions. Bacilli were fixed in 4% paraformaldehyde for 20 min and rinsed. According to manufacturer’s protocol, viability analysis was carried out by FACS separating bacilli in two regions of the log-integrated red fluorescence propidium iodide versus Syto 9; the number of bacilli found within these regions were used to estimate the percentage of viability (Fig. 2a). The results showed that at 1:10 MOI the viability of the Pi-deprived bacilli was significantly increased (Fig. 2b).

### High phagocytosis of microbeads coated with plasma membrane proteins of phosphate-deprived mycobacteria

**Fig. 3:**
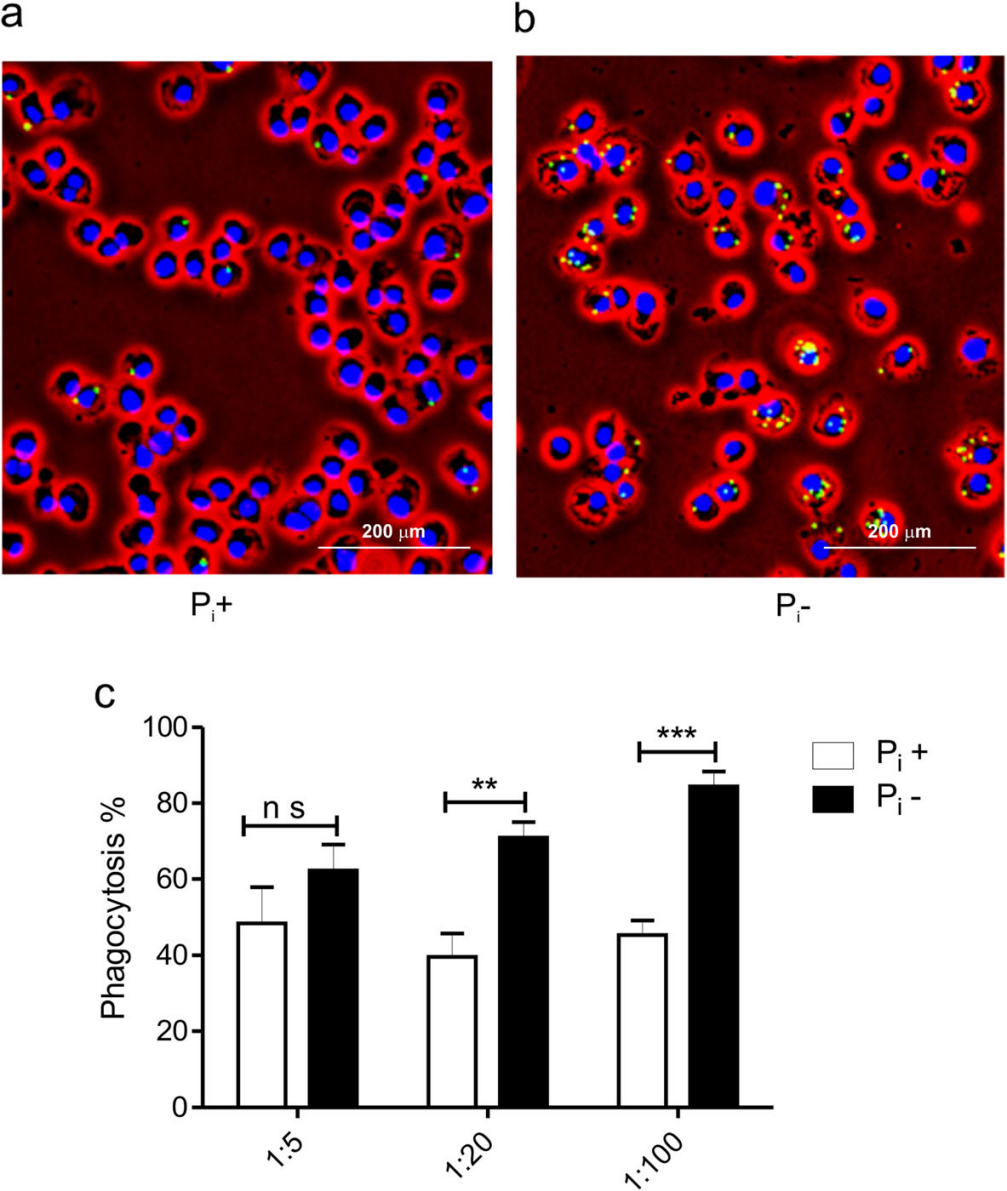
Phagocytosis of membrane protein-coated microbeads is higher with phosphate deprived bacilli proteins. Assays were carried out with J774A.1 macrophage-like cells and green fluorescent microbeads coated with plasma membrane proteins obtained from mycobacteria grown with and without Pi. MOs (5 × 10^5^) were placed on acid-washed glass coverslips. Microbeads were added to cells at a 1:5, 1:20 and 1:100 rate for 4 h at 37 °C in 5% CO_2_. The percentage of cells with ingested or bound microbeads was estimated counting at least 400 MOs (**a**, **b**). At 1:100 rate the phagocytosis of microbeads coated with proteins of Pi-deprived bacilli was 84.44% and of bacilli grown with Pi it was 45.4% (**a**, **c**). *p* < 0.05; unpaired t Student’s test. Results of 5 experiments are presented

To study the role of mycobacteria proteins in the phagocytosis of bacilli grown under Pi starvation conditions, we carried out assays with fluorescent microbeads coated with proteins obtained from plasma membranes isolated from whole mycobacteria grown with and without Pi. For phagocytosis assays, MOs (5 × 10^6^) cultured in RPMI 1640 with 10% heat-inactivated fetal bovine serum were placed on acid-washed glass coverslips. Microbeads were added to cells at 1:5, 1:20 and 1:100 rate for 4 h at 37 °C in 5% CO_2_. To quantitate phagocytosis of microbeads, the slides were analyzed with a Nikon epifluorescence microscope examining at least 400 cells in random fields of 3 slides at each experimental time (Fig. 3a, b). MOs were scored positive regardless of the number of bound or engulfed microbeads. Immunofluorescence microscopy of 400 or more cells showed that binding/phagocytosis of microbeads was dose-dependent and significantly higher with microbeads coated with membrane proteins obtained from bacilli grown without Pi (Fig. 3c); this difference was evident in assays incubating cells at 1:20 and 1:100 macrophage/microbead rates.

### Phagosome acidification in macrophages that have engulfed phosphate-deprived mycobacteria

**Fig. 4:**
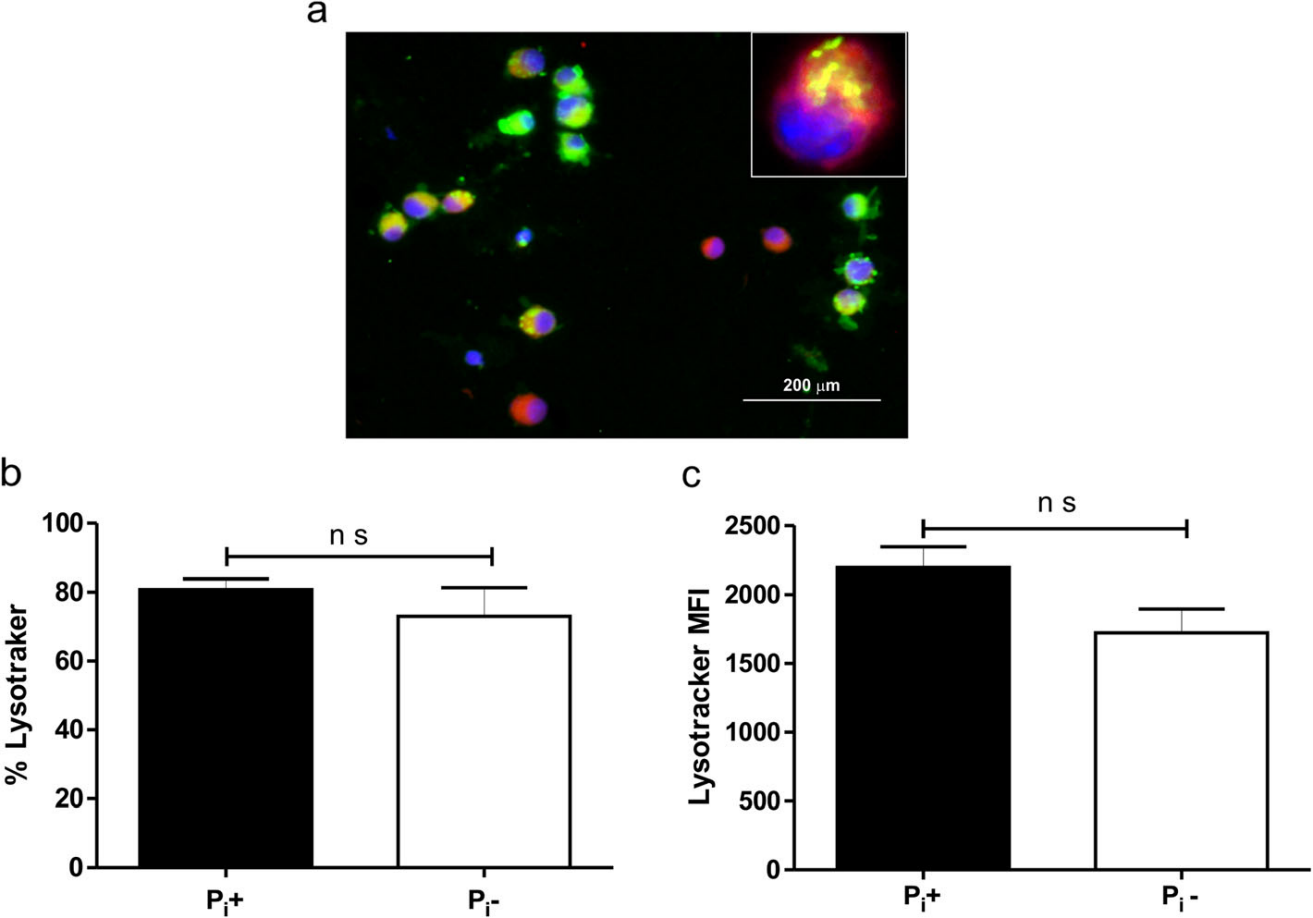
Phagosome acidification in MOs that engulfed mycobacteria grown without phosphate. Phagocytosis assays were performed with MOs labelled with LysoTracker, whose red fluorescence intensifies at low pH, and FITC-labelled bacilli cultured with or without Pi. By immunofluorescence microscopy at least 400 MO were counted. MO emitting red fluorescence were scored as Lysotracker positive. The immunofluorescence patterns are shown; bacilli located in non-acidified compartments (LysoTracker negative) are green fluorescent. In acidified vacuoles (LysoTracker positive) bacilli emitted yellow fluorescence due to overlapping of FITC-labelled bacilli and Lysotracker red fluorescence (insert), (**a**). It was found that 80.75% of MOs incubated with mycobacteria grown with usual amount of Pi were LysoTracker positive while incubation with Pi-deprived bacilli 76.06% of MO were Lysotracker labelled (**b**), a difference that was not statistically significant (n s; Mann-Whitney Test). Four independent experiments were performed. FACS showed that the mean fluorescence index (MFI) was less in MO that phagocytosed Pi-deprived mycobacteria (**c**). This difference was not statistically significant (n s; Mann-Whitney Test)

An outstanding ability of virulent mycobacteria is to inhibit phagosome acidification and phagolysosome fusion [2]. We studied the phagosomal acidification process by immunofluorescence microscopy using *M. bovis*/BCG grown with or without Pi labelled green with fluorescein isothiocyanate (FITC) and MOs labelled with LysoTracker, a marker that turns red at low pH (Fig. 4a). Bacilli located in Lysotracker positive vacuoles were yellow fluorescent due to overlapping of FITC and the red fluorescence of Lysotracker. The engulfed bacilli emitted a FITC green fluorescence when were located in Lysotracker negative vacuoles (Fig. 4a). When infected with bacilli grown with usual amounts of Pi, 80.75% of cells were Lysotracker positive and with Pi-deprived bacilli the percent of Lysotracker labeled MOs was reduced to 73.06% FACS analysis of the phagocytosis assays confirmed the immunofluorescence findings showing that Pi-deprived bacilli phagocytosis is associated with decreased Lysotracker labeling (Fig. 4b, c).

### Phosphate-starved mycobacteria increment the expression of adhesins in isolated plasma membranes

**Fig. 5:**
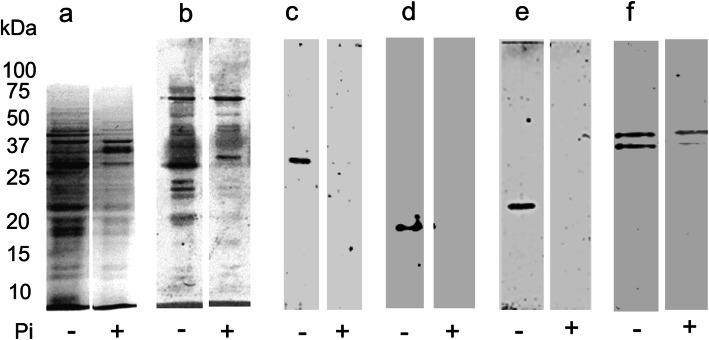
Cell membrane located PstS-1, LpqH, LprG, and APA are expressed by Pi-deprived mycobacteria. *M.bovis*/BCG bacilli were cultured with (+) and without Pi (−) for 24 h and the membranes were isolated by sonication and centrifugation, separated by 12% SDS-PAGE and stained with Coomasie blue (**a**). The membranes were transferred to PVDF for immunoblot with an anti-*M.bovis*/BCG antiserum which detected many protein bands more numerous in membranes of Pi-deprived bacilli (**b**). Using Abs available in our laboratory the expression of PstS-1, LpqH, LprG, and APA was demonstrated in isolated plasma membranes of Pi-deprived mycobacteria (**c**-**f**)

We have previously shown by immunoelectron microscopy that PstS-1 is a cell surface located adhesin that triggers the phagocytosis of mycobacteria [10]. To study its location in Pi- starved mycobacteria, we isolated plasma membranes to carry out 1-D electrophoresis and immunoblot (Fig. 5). By Coomasie blue staining and immunoblot with a polyclonal anti-*M. bovis*/BCG antiserum the number of protein bands was higher in membranes of Pi-deprived mycobacteria. In the isolated plasma membranes immunobloting with mAb available in our laboratory that recognizes adhesins that participate in the phagocytosis of mycobacteria 5 reactive bands of 38, 19, 23, and 47 kDa that correspond to PstS-1, LpqH, LprG, and the APA antigen, respectively (Fig. 5). In the membranes of bacilli grown with Pi band mild APA reactivity was observed.

## Discussion

In this study, we observed that *M. bovis*/BCG cultured without Pi acquire properties that could indicate increased virulence. First, bacilli were phagocytosed by MOs with high efficiency, a property suggesting increased infectivity. These changes were associated with an increase in cell protein band expression in cell membranes. Morever, in isolated plasma membranes of Pi-deprived bacilli, it was demonstrated the expression of glycoproteins that have been characterized as adhesins that play a critical role in the infection of host cells.

It is noteworthy that mycobacteria subjected to the metabolic stress implied in Pi deprivation increment the expression of adhesins which are virulence molecules that bind to complementary molecules present in the surface of MOs and other host cells [11]. Information on the repertoire of Mtb adhesins is needed since it could help to design prevention and treatment strategies aimed to block the crucial step that is the infection of host cells by the mycobacteria. A few mycobacterial adhesins have been characterized including PstS-1, the 38-kDa antigen which is high-affinity protein that transports Pi [10, 13]. It has been recognized its high antigenicity at the humoral level that allowed the design of diagnostic serologic tests [16–19]. PstS-1 was characterized as a glycoprotein with mannose residues and more recently its role of adhesin in the phagocytosis of mycobacteria was recognized [10]. PstS-1 has focused much interest as a high-affinity Pi transporter that carries Pi from the periplasm to the cytoplasm. It has been reported that Pi-starved mycobacteria upregulates PstS-1 which is 30% similar in amino acid sequence to PstS-1 from *Escherichia coli* [12].

Our current findings suggesting increased virulence in mycobacteria subjected to Pi deprivation are in keeping with previous studies [20]. Pst genes are overexpressed in mycobacteria grown under Pi starvation conditions; mutations in these genes affect the import of Pi and reduces the proliferation of mycobacteria inside macrophages and in experimentally infected mice [21, 22]. On the other hand, it has been observed that Mtb Pst system neutralizes IFN-gamma-dependent host immunity [22].

There is much information about the role membrane proteins of Mtb and other microbes play in the pathogenesis of infection [23]; their strategic location at the microbe surface allow their efficient interaction with host cells to promote phagocytosis and when they are antigenically active an immune response that can be cell or antibody-mediated [23]. Mycobacterial membrane proteins may also exert inhibitory effects on host cell activity. In this study we found that in addition to PstS-1, Pi-deprived mycobcteria express membrane proteins that are considered adhesins involved in the pathogenesis of the mycobacterial infection. LpqH, the 19 kDa glycolipoprotein, participates in MO infection as an adhesin that binds the mannose receptor triggering the phagocytosis of bacilli and downregulating MHC-II antigen presentation interfering with TLR2 and MAPK signaling [22]. The APA antigen plays a prominent role in Mtb virulence; it functions as a mycoyl-transferase that catalyzes the attachment of mycolic acids to arabinogalactan and the biogenesis of cord factor, a very active virulence factor [24]. APA is expressed at the bacterial surface, which explains its interaction with fibronectin, elastin and the surfactant protein A. LprG, the 25-kDa glycolipoprotein transports ManLAM to the mycobacterial cell-wall and as an adhesin it may bind the MO mannose receptor facilitating phagocytosis and inhibition of the phagosome-lysosome fusion. Isolated LprG was found to inhibit MHC-II Ag processing by THP-1 cells and primary human MO through prolonged TLR2 activation [25, 26].

## Conclusions

The response of M. bovis/BCG to Pi deprivation could be of interest for vaccine design. The increased phagocytosis and intracellular viability of phosphate deprived bacilli could favor the activation of immunocompetent cells. The fact that phagosome acidification is virtually preserved would favor the lysis of mycobacteria and the generation of antigenic peptides which are needed to induce an effective immune response. To gain insight in the meaning of our findings, studies with dendritic cells, the master antigen presenting cell, are required.

## Methods

### Antibodies

Monoclonal antibodies to LpqH and PstS-1 were obtained from BEI Resources (Manassas, VA, USA). A monoclonal antibody to Apa was developed in our laboratory. A polyclonal antiserum to LprG was donated by Dr. Clara Espitia (Universidad Nacional Autónoma de México, Mexico City).

### Culture of *Mycobacterium bovis*/BCG under phosphate deprivation conditions

The method of Braibant et al. was followed [14]. Briefly, *M. bovis*/BCG was cultured in Sauton medium with K_2_HPO_4_ (0.5 g/L) at 37 °C, until optic density of 460 was reached. Thereafter, aliquots were taken and inoculated in Sauton medium that was supplemented with an excess of K_2_HPO_4_ to a final concentration of 3 g/L; the mycobacteria were cultured at 37 °C until optic density of 460 was reached. After that, two aliquots were rinsed, centrifuged at 6000 g, and inoculated in Sauton medium. One aliquot was cultured without K_2_HPO_4_ and the other with 0.5 g/L K_2_HPO_4_. After 24 h of culture, bacilli were collected, rinsed with TBS and frozen until use.

### Phagocytosis assays with phosphate-deprived mycobacteria

We carried assays with mycobacteria that were grown with and without Pi as described above. The Balb/c-derived murine macrophage-like tumor cell line J774A.1 was obtained from the American Type Culture Collection (Manassas, VA, USA). Cells were cultured in RPMI 1640 supplemented with 10% fetal calf serum. MOs (5 × 10^5^) were incubated with PKH26 labeled bacilli at 1:2, 1:5, and 1:20 MOI, at 37 °C for 4 h. After extensive rinsing with PBS, the cells were fixed in 4% paraformaldehyde in PBS for 20 min. For immunofluorescence microscopy, cytospin slides were mounted with ProLong Antifade with DAPI (Invitrogen, Eugene, OR, USA) and examined with a Nikon epifluorescence microscope. Phagocytosis was analyzed by FACS. At least 10,000 cells were analyzed in a FACSClibur (Becton Dickinson, San Jose CA, USA) operating with FlowJo 7 software and a 488 nm argon laser. The gates were set following established forward and side scatter parameters.

### Analysis of the mycobacterial viability after phagocytosis

Assays to verify the viability of phagocytosed mycobacteria were carried out. After 4 h phagocytosis of mycobacteria at 1:10 and 1:20 MOIs, MOs were recovered in TBS, lysed with 0.1% sodium deoxycholate for 5 min, and centrifuged twice at 12,000 rpm for 10 min. Thereafter, to assess viability the LIVE/DEAD Baclight bacterial viability Kit (Molecular Probes) was used following the manufacturer instructions. Afterward, bacilli were fixed in 4% paraformaldehyde for 20 min, rinsed in TBS and analyzed by flow cytometry. Because both live and dead cells exhibit green fluorescence, the log-integrated green fluorescence was adjusted to eliminate debris. Populations of bacteria were discriminated as two regions of the log-integratedred fluorescence, and the numbers of bacteria found within these regions were used to estimate the percentage of viable organisms in the population.

### Binding/phagocytosis assays with microbeads coated with mycobacterial membrane proteins

We assessed a possible role of mycobacterial membrane proteins in the phagocytosis of mycobacteria employing green fluorescent Polystyrene 1 μm microbeads (Molecular Probes, Eugene, OR, USA) coated with plasma membrane proteins obtained from mycobacteria grown with and without Pi. Microbeads and 500 μg membrane proteins were mixed in 1 mL MES Buffer 2-[N-morpholino] ethane sulfonic acid (pH 6). After that, 1-ethyl-3-(3-dimethylamine nopropyl)-carbodiimide was added to a 2.5/ml final concentration. After overnight incubation, 100 mM glycine was added to stop the reaction. To block remaining reactive sites, microbeads were rinsed with PBS and kept at 4 °C in PBS containing 1% BSA. For phagocytosis assays, 5 × 10^5^ MOs were cultured in RPMI 1640 with 10% heat-inactivated FBS and placed on acid-washed glass coverslips. Microbeads were added to cells at a 1:20 rate and incubated for 4 h at 37 °C in 5% CO_2_. The slides were rinsed in PBS to remove non-adherent microbeads and fixed in 1% paraformaldehyde for 10 min.

### Phagosome maturation after phagocytosis of mycobacteria

For these analyses bacilli grown with and without PI were labeled green with fluorescein isothiocyanate (FITC). Briefly, 50 μl of a bacilli suspension was suspended in 950 μl carbonate buffer 0.1 M, pH 9 and 0.5 mg FITC were added for 2 h. To finish labeling the mycobacteria were centrifuged at 10,500 g for 10 min and rinsed 3 times in PBS. To examine whether Pi deprived mycobacteria acquired the ability to alter phagosome maturation, we use the acidotropic dye LysoTracker Red DND-99 (Molecular Probes, Eugene, OR). After rinsing, FITC labeled mycobacteria were added for 4 h at a 1:10 MOI. In a final step, LysoTracker was added in RPMI (1:20,000) for 20 min before phagocytosis was finished. MOs were rinsed, fixed with 4% paraformaldehyde and placed in glass slides. To verify LysoTracker labeling slides were examined by immunofluorescence microscopy. Besides, we quantitated by FACS the extent of LysoTracker phagosome labeling and the phagocytosis of FITC labeled bacilli.

### Isolation of mycobacterial plasma membranes to identify proteins expressed during phosphate deprivation

Mycobacteria were grown with and without Pi as described above. To obtain mycobacterial plasma membranes we followed a published method [27]. Briefly, in a lysis buffer containing protease inhibitors, bacilli were sonicated at 60 kHz in iced water (20 cycles 1 min each) and centrifuged at 1000 g and the supernatant was centrifuged at 27, 000 g; the supernatant obtained was centrifuged at 100,000 g to obtain in the pellet the plasma membranes. Protein concentration was measured by the Lowry protein assay (Bio-Rad Laboratories, Hercules, CA, USA). To identify membrane proteins were separated by 12% SDS-PAGE, stained with Coomassie blue, transferred to PVDF membranes and incubated with a rabbit antiserum to *M. bovis*/BCG. Isolated plasma membranes were identified with a mAb to LpqH and PstS-1 (donated by BEI Resources (Manassas, VA, USA), and to APA (developed in our laboratory) and with a polyclonal antiserum to LprG (donated by PhD Clara Espitia); bound antibodies were detected with goat anti-mouse and a goat anti-rabbit IgG antibodies labeled with horseradish peroxidase. The reactive bands were visualized by chemiluminescence with SuperSignal West Dura kit (Pierce, Rockford, IL, USA).

## Data Availability

All data are included in the manuscript.

## Abbreviations

TB: Tuberculosis
Mtb: *Mycobacterium tuberculosis*
BCG: Mycobacterium bovis Calmette Guerin
Pi: Phosphate
FACS: Fluorescence activated cell sorter
MFI: Mean fluorescence index
FITC: Fluorescein isothiocyanate
